# *Human cytomegalovirus* may promote tumour progression by upregulating arginase-2

**DOI:** 10.18632/oncotarget.9722

**Published:** 2016-05-30

**Authors:** Helena Costa, Xinling Xu, Gitta Overbeek, Suhas Vasaikar, C. Pawan K. Patro, Ourania N. Kostopoulou, Masany Jung, Gowhar Shafi, Sharan Ananthaseshan, Giorgos Tsipras, Belghis Davoudi, Abdul-Aleem Mohammad, Hoyin Lam, Klas Strååt, Vanessa Wilhelmi, Mingmei Shang, Jesper Tegner, Joo Chuan Tong, Kum Thong Wong, Cecilia Söderberg-Naucler, Koon-Chu Yaiw

**Affiliations:** ^1^ Cell and Molecular Immunology, Department of Medicine, Center for Molecular Medicine, Unit for Experimental Cardiovascular Research and Department of Neurology, Karolinska Institutet, Stockholm, Sweden; ^2^ Unit of Computational Medicine, Department of Medicine, Center for Molecular Medicine, Karolinska Institutet, Stockholm, Sweden; ^3^ Social & Cognitive Computing Department, Institute of High Performance Computing, Agency for Science, Technology and Research, Singapore; ^4^ Department of Genomics and Bioinformatics, Positive Bioscience, Mumbai, India; ^5^ Division of Gene Technology, School of Biotechnology, Science for Life Laboratory, Royal Institute of Technology (KTH), Solna, Sweden; ^6^ Department of Pathology, Faculty of Medicine, University of Malaya, Malaysia; ^7^ Present affiliation: Division of Cancer Studies, King's College London, London, UK

**Keywords:** cytomegalovirus, glioblastoma, arginase, treatment

## Abstract

**Background:**

Both arginase (ARG2) and *human cytomegalovirus* (HCMV) have been implicated in tumorigenesis. However, the role of ARG2 in the pathogenesis of glioblastoma (GBM) and the HCMV effects on ARG2 are unknown. We hypothesize that HCMV may contribute to tumorigenesis by increasing ARG2 expression.

**Results:**

ARG2 promotes tumorigenesis by increasing cellular proliferation, migration, invasion and vasculogenic mimicry in GBM cells, at least in part due to overexpression of MMP2/9. The nor-NOHA significantly reduced migration and tube formation of ARG2-overexpressing cells. HCMV immediate-early proteins (IE1/2) or its downstream pathways upregulated the expression of ARG2 in U-251 MG cells. Immunostaining of GBM tissue sections confirmed the overexpression of ARG2, consistent with data from subsets of Gene Expression Omnibus. Moreover, higher levels of ARG2 expression tended to be associated with poorer survival in GBM patient by analyzing data from TCGA.

**Methods:**

The role of ARG2 in tumorigenesis was examined by proliferation-, migration-, invasion-, wound healing- and tube formation assays using an ARG2-overexpressing cell line and ARG inhibitor, N (omega)-hydroxy-nor-L-arginine (nor-NOHA) and siRNA against ARG2 coupled with functional assays measuring MMP2/9 activity, VEGF levels and nitric oxide synthase activity. Association between HCMV and ARG2 were examined in vitro with 3 different GBM cell lines, and ex vivo with immunostaining on GBM tissue sections. The viral mechanism mediating ARG2 induction was examined by siRNA approach. Correlation between ARG2 expression and patient survival was extrapolated from bioinformatics analysis on data from The Cancer Genome Atlas (TCGA).

**Conclusions:**

ARG2 promotes tumorigenesis, and HCMV may contribute to GBM pathogenesis by upregulating ARG2.

## INTRODUCTION

Arginase is a metalloenzyme that hydrolyzes the semi-essential amino acid L-arginine to L-ornithine and urea. L-ornithine is the precursor for L-proline (an essential component of collagen) and polyamines that are involved in cellular proliferation, differentiation, and neoplastic transformation [1]. The metabolism of L-arginine to L-ornithine is primarily based on arginase 2 (ARG2) [2], an isoform of arginase which differs from the ARG1 isoform in structure, cellular location, and function [3]. Elevated levels of ARG1 and/or ARG2 expression have been implicated in immune cell dysfunction, infections, vascular diseases and many types of cancers (primarily ARG1) [4–9]. For instance, in Hepatitis C virus (HCV)-driven hepatocarcinogenesis, induction of ARG1 by HCV may play a crucial role by increasing the availability of ornithine and polyamines, which could promote the growth and survival of HCV-infected hepatocytes [9]. However, the role of ARG2 in the pathogenesis of glioblastoma (GBM) is unknown.

GBM is the most common malignant and lethal brain tumor with 12-15 months median survival with standard treatment despite advances in the treatment options [10]. *Human cytomegalovirus* (HCMV), a ubiquitous beta herpesvirus, has been implicated in the pathogenesis of many chronic and inflammatory diseases and in cancer, particularly GBM [11–14]. We recently showed that HCMV immediate-early (IE) proteins may induce vasculopathy by inducing ARG2 expression in endothelial cells [6]. Pathogenic HCMV IE proteins also orchestrate the virus-host interactions as well as viral replication, and thus are central to viral pathogenesis [15]. Given the tumorigenic property of aberrant arginase expression and probable role of HCMV in carcinogenesis, we hypothesize that HCMV could contribute to tumorigenesis by modulating arginase expression.

## RESULTS AND DISCUSSION

### Overexpression of ARG2 in U-251 MG cells indicates promotion of proliferation, migration, invasion and vasculogenic mimicry

**Figure 1:**
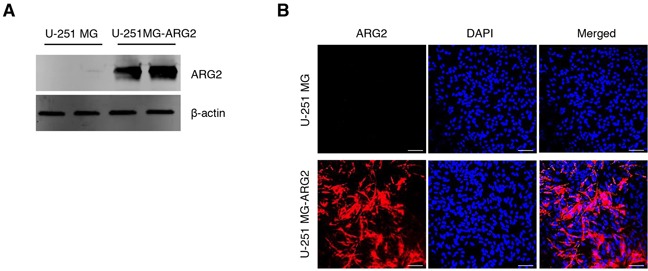
Characterization of the stable cell line U-251 MG-ARG2 Stable overexpression of ARG2 in U-251 MG cells was confirmed by **A.** western blot and **B.** immunofluorescence using ARG2 specific antibody. Nuclei were stained with DAPI (blue). Size bar: 100 μm.

**Figure 2:**
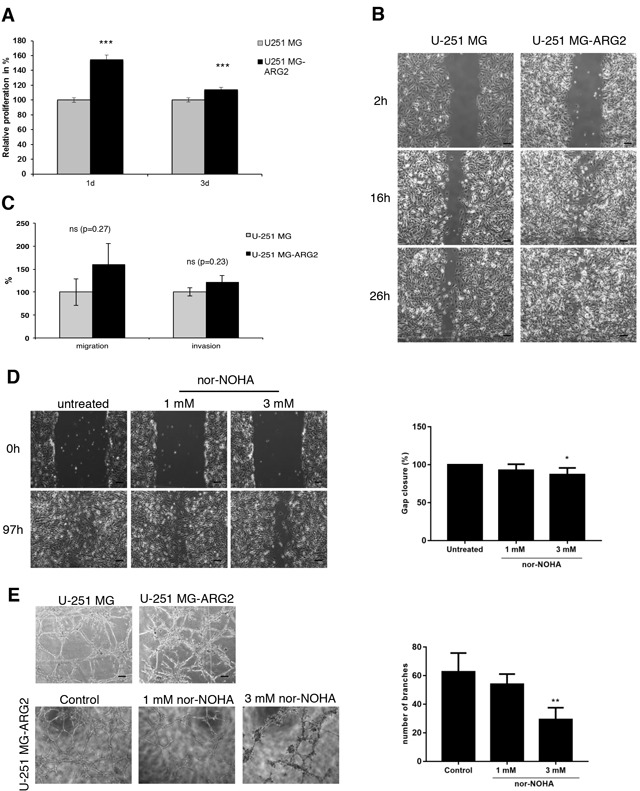
Effect of stable overexpression of ARG2 on proliferation, migration and tube formation of U251 MG cells **A.** Proliferation of U-251 MG-ARG2 cells was analyzed by MTT assay and normalized to parental U-251 MG cells. Bars are mean±SD (*n*=8) **B.** Representative light microscopic images of the wound healing assay at different times after the “scratch” of U-251 MG or U-251 MG-ARG2 cell monolayers. Size bar: 50 μm **C.** Migration and invasion of U-251 MG-ARG2 cells were quantified and normalized to parental U-251 MG cells at 24h and 48h, respectively. Data represent mean±SD (*n*=2) **D.** Representative light microscopic images of the wound healing assay at different times after the “scratch” of U-251 MG-ARG2 cell monolayer in the absence or presence of the ARG2 inhibitor nor-NOHA. Size bar: 50 μm. Quantification of gap closure 97h after treatment with different doses of nor-NOHA is shown. Data represent mean±SD of five randomly selected distances **E.** Representative light microscopic images of tube formation by U-251 MG or U-251 MG-ARG2 cells 6d after seeding (upper panel) or by U-251 MG-ARG2 untreated or treated with different doses of nor-NOHA (lower panel) (N=3). Quantification of the number of branches formed under the different conditions is shown on the right. Size bar: 50 μm. * p < 0.05, **p < 0.01, ***p < 0.001.

To better understand the role of ARG2 in GBM pathogenesis, we generated and verified a stable cell line overexpressing ARG2, which we named U-251 MG-ARG2 (Figure 1). A standard MTT assay of cell metabolic activity showed that U-251 MG-ARG2 had a higher metabolism than the parental U-251 MG cells, evident as early as 1 day after seeding (Figure 2A). Furthermore, U-251 MG-ARG2 had an increased migratory property (Figure 2B). Using a commercially available migration and invasion kit, we found that the U-251 MG-ARG2 tended to have a higher invasion and migration activity than the parental cells albeit statistically not significant (Figure 2C). Treatment with arginase inhibitor nor-NOHA at 3 mM, significantly reduced the migratory property of the U-251 MG-ARG2 cells (Figure 2D, right panel).

**Figure 3:**
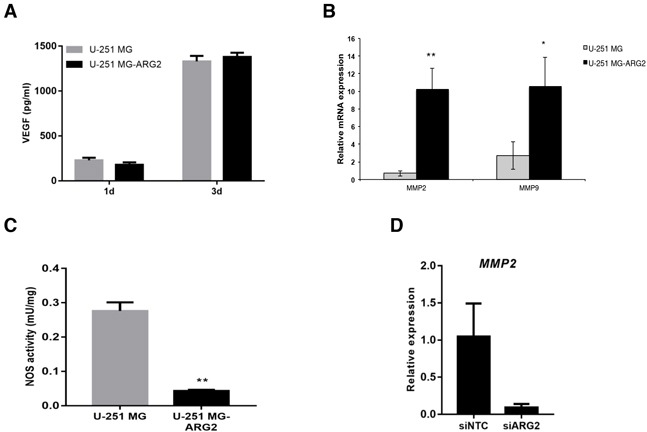
**A.** Levels of VEGF in supernatants of U-251 MG and U-251 MG-ARG2 were quantified by ELISA after 1 and 3 days in culture. **B.** Expression of MMP2 or MMP9 in U-251 MG-ARG2 cells relative to parental U-251 MG cells was determined by qPCR. Beta 2-microglobulin was used as endogenous control. **C.** NOS activity was detected in U-251 MG and U-251 MG-ARG2. **D.** Relative expression of ARG2 in U-251 MG transfected with control or ARG2 specific siRNA. Bars represent mean±SD (*n*=3). * *p* < 0.05, **p < 0.01, ***p < 0.001.

Since angiogenesis plays a crucial role in tumorigenesis, we assessed the vasculogenic mimicry of the cells by a standard tube formation assay. The optimal cell number for parental and U-251 MG-ARG2 cells was determined prior to the experiment (data not shown). U-251 MG-ARG2 produced more and shorter branches than the parental cells (Figure 2E, top panel) and treatment with nor-NOHA at 3 mM significantly inhibited the proper tube formation (Figure 2E, bottom panel and right panel) in the U-251 MG-ARG2 cells. Both anti-migratory and tube formation effects by nor-NOHA were not due to cytotoxic effect of nor-NOHA used at 3 mM (Supplementary Figure S1). In addition, the increased angiogenic property of U-251 MG-ARG2 was not due to VEGF, at least at the assayed time point (Figure 3A). Thus, overexpression of ARG2 in U-251 MG renders the cells pro-angiogenic.

To further dissect possible mechanisms for the increased tumorigenic property of U-251 MG-ARG2, we quantified the mRNA expression of matrix metalloproteinase MMP2 and MMP9, which have been linked to tumor progression, invasion and angiogenesis [16, 17]. Both MMPs were expressed at higher levels in U-251 MG-ARG2 than in the parental cells (Figure 3B). Thus, the increased vasculogenic mimicry or invasiveness of U-251 MG-ARG2 cells may be due to overexpression of MMP2, MMP9 or both but was unlikely to be due to activation of nitric oxide synthase (Figure 3C). To further corroborate the role of ARG2 in modulating the expression of MMP2/9, we investigated the effects of siRNA on ARG2 expression. Silencing of ARG2 reduced significantly MMP2 expression levels in the U-251 MG cells, while we did not detect MMP9 at the basal level, suggesting ARG2 can directly modulate MMP2 expression (Figure 3D). By bioinformatics analysis of MMP2 and MMP9 expression in a mouse model of glioma, we observed 2 out of 6 cases with higher MMP2 expression, and 3 cases with higher MMP 9 expression vs. controls (Supplementary Figure S2). These results indicate high variability between individual cases. Collectively, these findings demonstrate that ARG2 may promote tumorigenesis by increasing cell proliferation/metabolism, migration, invasion and vasculogenic mimicry via MMP2/9 modulation.

### HCMV upregulates ARG2 expression

**Figure 4:**
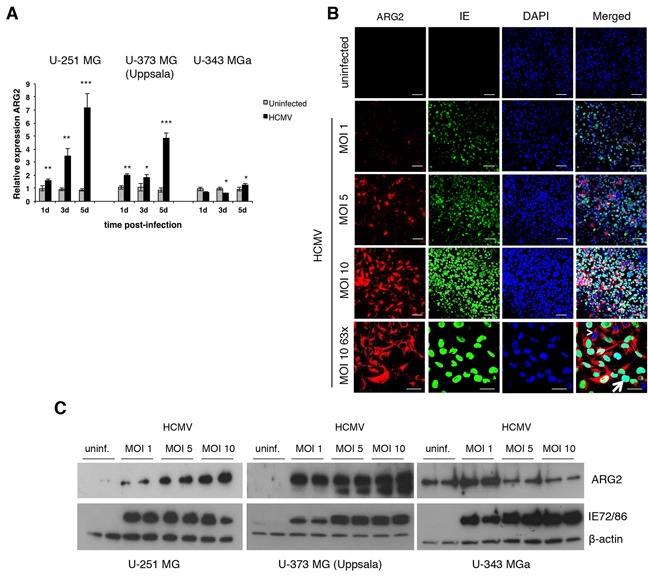
HCMV upregulates ARG2 in glioblastoma cell lines **A.** Expression of ARG2 in HCMV-infected (MOI 5) U-251 MG, U-373 MG (Uppsala) and U-343 MGa cell lines relative to uninfected cells determined by qPCR at 1, 3, and 5 dpi. Beta 2-microglobulin was used as endogenous control. Bars represent mean±SD (*n*=3). *p<0.05, **p<0.01, ***p<0.001 **B.** Representative images of immunofluorescent staining for ARG2 (red) and HCMV IE (green) in uninfected and different multiplicity of infection (MOI) of HCMV-infected U-251 MG cells at 5 dpi. Nuclei were stained with DAPI (blue). Arrowhead: HCMV IE negative cell expressing ARG2. Arrow: HCMV IE positive cell. Size bar: 100 μm (top and second row), 50 μm (bottom row). **C.** Protein levels of ARG2 in uninfected and HCMV-infected U-251 MG, U-373 MG (Uppsala) and U-343 MGa cells (duplicates) were determined by western blot at 5 dpi. Detection of HCMV IE72/86 proteins was used as infection control and β-actin as loading control.

To further assess a potential role of HCMV in tumorigenesis, we infected three GBM cell lines (U-251 MG, U-373 MG (Uppsala) and U-343 MGa) with HCMV and measured both ARG1 and ARG2 mRNA expression by quantitative TaqMan PCR assay. Since we could only detect low levels of ARG1 expression in U-373 MG (Uppsala) but not in U-251 MG and U-343 MGa cells; in addition, HCMV has no effect on ARG1 expression (unpublished data), therefore we focused on ARG2. At baseline, ARG2 mRNA expression was the highest in U-343 MGa among 3 cell lines (Figure S3). The viral infection induced ARG2 expression in U-251 MG and U-373 MG (Uppsala) cells over time, especially at 5 days post-infection (dpi) (Figure 4A). However, in U-343 MGa cells, ARG2 expression was first suppressed at 1- and 3-dpi before a trend for higher expression occurred at 5-dpi (Figure 4A). The virus-induced ARG2 expression was further corroborated by immunofluorescent staining and western blot (Figure 4B and 4C, respectively). Of note, not all HCMV IE-positive cells were positive for ARG2 (Figure 4B, panel MOI 10 63x, arrow), which suggests that a threshold infection level is necessary for ARG2 induction, and ARG2 expression was detected in the neighboring uninfected cells (Figure 4B, panel MOI 10 63x, arrowhead), suggesting a paracrine effect perhaps dependent on a concentration gradient of cytokines released by infected cells (Supplementary Figure S4). Thus, HCMV infection induces ARG2 expression in U-251 MG and U-373 MG (Uppsala) cells, but reduced ARG2 protein expression in U-343 MGa cells at MOI of 5 and 10 (Figure 4C and S5). Of note, U-343 MGa has the highest infectious virus particles in the supernatants (Supplementary Figure S6). These differences in ARG2 expression may mirror the heterogeneity of the GBM cell lines (as also revealed by our bioinformatics analysis with data from The Protein Atlas, see Supplementary Figure S7) and their different responses to HCMV infection.

### HCMV IE proteins or their downstream pathways upregulate ARG2 expression in U-251 MG cells

**Figure 5:**
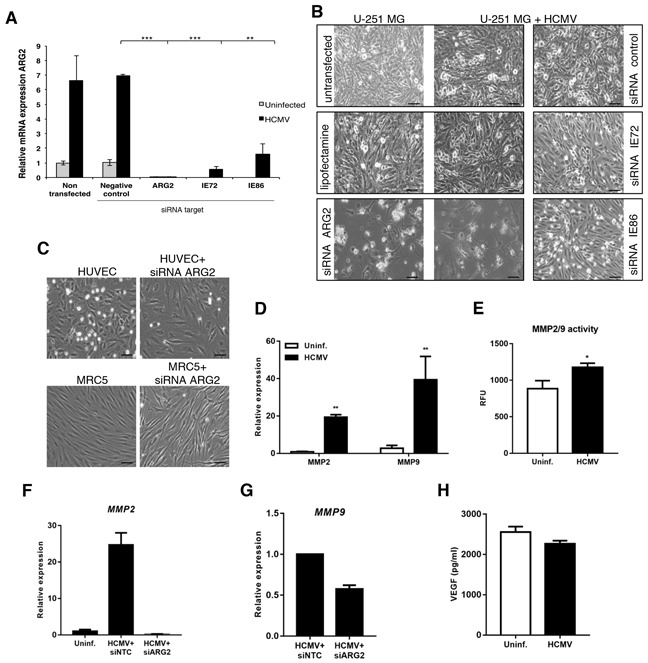
HCMV upregulates ARG2 in glioblastoma cell lines through the expression of IE genes **A.** Relative expression of ARG2 in uninfected or HCMV-infected U-251 MG cells transfected with negative control siRNA or specific siRNAs targeting ARG2, IE72 or IE86 determined by qPCR. 18S rRNA was used as an endogenous control. **B.** Representative phase contrast images of U-251 MG non-transfected or transfected with different siRNAs targeting ARG2, IE72 or IE86 in the presence or absence of HCMV, 4dpi. Lipofectamine alone and siRNA control were used as negative control. Size bar: 100 μm **C.** Phase contrast images of HUVEC and MRC-5 cell lines non-transfected or transfected with siRNA targeting ARG2. Size bar: 100 μm. **D.** Relative expression of MMP2 and MMP9 in U-251 MG cells infected with HCMV at 5dpi was determined by qPCR. **E.** MMP2/9 (gelatinase) activity was determined in U-251 MG cells uninfected or infected with HCMV at 5dpi. RFU, relative fluorescent units **F, G.** Relative expression of MMP2 or MMP9, respectively, in U-251 MG cells infected with HCMV and transfected with control or ARG2 specific siRNA. **H.** Levels of VEGF in supernatants of U-251 MG uninfected or infected with HCMV were quantified by ELISA at 5dpi. Bars represent mean±SD (n=3). *p<0.05, **p<0.01, ***p<0.001.

To gain further mechanistic insight into the viral regulation of ARG2 expression, we performed siRNA experiments in U-251 MG cells, as they had the greatest increase in ARG2 mRNA expression upon HCMV infection (see Figure 4A). Silencing of both IE72 and IE86, which are critical for viral replication and pathogenesis, reduced expression of ARG2 mRNA by 92% and 76%, respectively (Figure 5A). The ARG2 silencing efficiency was 98.2 ± 1.2% (range 96.9-99.4%, n=3); negative control siRNA had little or no effect on ARG2 expression (Figure 5A). Of note, silencing ARG2 caused substantial lysis of both uninfected and HCMV-infected U-251 MG cells, whereas little or no lysis was noted in equivalent cells transfected with IE72, IE86 or negative control siRNA (Figure 5B). Moreover, silencing ARG2 in primary endothelial cells or fibroblasts did not result in cell lysis (Figure 5C). These results suggest that ARG2 is crucial for the survival of U-251 MG cells. To further link HCMV infection with MMP2/9, we infected U-251 MG cells with HCMV and assayed their expression and activity with qPCR and ELISA, respectively at 5 dpi (Figure 5D-5E). Infection with HCMV induced both MMP2 and MMP9 expression and their activity (Figure 5D and 5E, respectively). Interestingly, silencing of ARG2 in HCMV-infected U-251 MG abolished the induction of MMP2 and MMP9 by HCMV to the basal level (Figure 5F and 5G, respectively), suggesting ARG2 modulates directly the MMP2/9 expression. However, no difference was noted in the levels of VEGF with or without HCMV infection (Figure 5H) and HCMV infection resulted in reduction of NOS activity (Supplementary Figure S8).

### ARG2 is overexpressed in GBM tissues

**Figure 6:**
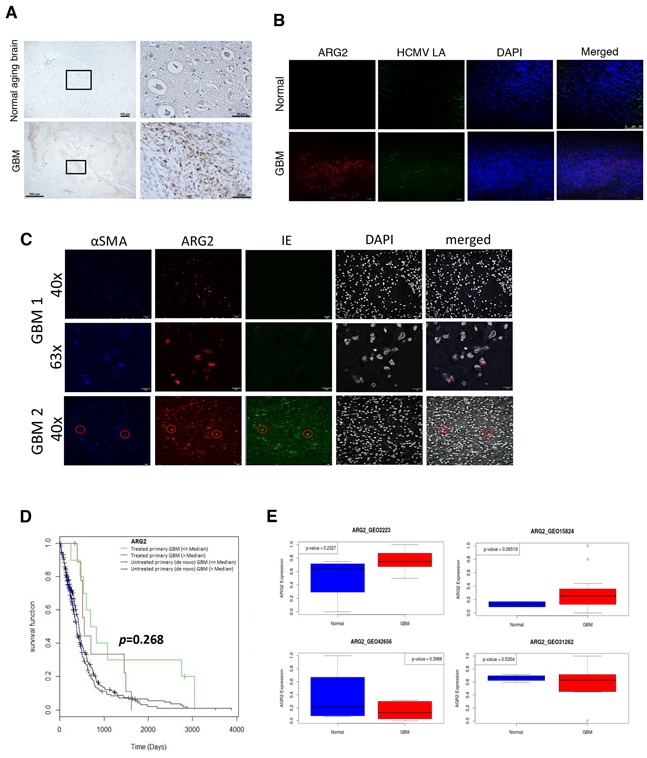
Expression of ARG2 in GBM tissue **A.** ARG2 was analyzed in tissue sections of normal brain and GBM by immunohistochemistry. Brown staining shows ARG2 positive cells. Size bar: 100 μm (upper left image), 500 μm (lower left image), 50 μm (images to the right). **B.** Immunofluorescent co-staining for HCMV late antigens (LA) and ARG2 in sections of normal brain and GBM. DAPI was used as a nuclear marker. Size bar: 50 μm. **C.** Immunofluorescent co-staining for alpha smooth muscle actin αSMA, HCMV IE and ARG2 in sections of GBM from two patients.DAPI was used as a nuclear marker. **D.** Correlation between ARG2 expression and survival in untreated and treated GBM patients using data from TCGA. Red and blue lines represent patients with ARG2 expression above median. **E.** ARG2 mRNA expression in control and GBM patients based on Gene Expression Omnibus (GEO) database.

To confirm our in vitro findings, we analyzed ARG2 protein expression with immunohistochemistry staining in formalin-fixed, paraffin-embedded GBM tissue specimens from 20 patients and 10 specimens of ‘normal’ aging brain. Nine of 20 (45%) tumor specimens were ARG2-positive, but all of the normal brain specimens were ARG2 negative. The ARG2 staining was cytoplasmic, dot-like or coarsely granular, and detected mostly in the periphery of tumor cells (Figure 6A). Importantly, dual immunofluorescent staining revealed ARG2 upregulation in HCMV-immunoreactivity cells (Figure 6B). However, not all ARG2-immunoreactivity cells were ‘co-localized’ with viral antigens and vice versa, suggesting other intrinsic factor(s) may be involved in the ARG2 upregulation. Our immunofluorescent staining showed that these cells were also stained for alpha smooth muscle actin and presumed to be cancer-associated fibroblasts (CAF) (Figure 6C), as judged by the similarities in morphology and staining pattern to those reported in pancreatic ductal carcinoma [7]. Of note, the ARG2-immunoreactive CAFs in that study were attributed to tissue hypoxia and associated with a shorter overall survival [7]. In a previous study, ARG2-expressing CAFs were associated with greater infiltration of CD68-positive macrophages and neutrophils but with lower infiltration of CD4+ and CD8+ T cells, indicating an immunosuppressive microenvironment [7]. The ARG-associated immunosuppression has also been reported recently by others [18]. As HCMV like other viruses is prone to be controlled by T-cell mediated immune response, the ‘ARG2-created immunosuppressive’ environment will thus favor virus survival. In addition, HCMV may induce ARG2 expression as part of its immune modulation. Moreover, HCMV is harbored in granulocyte-macrophage progenitors [19], whose differentiation into macrophages is closely related to reactivation of the virus [20].

Gene expression data from The Cancer Genome Atlas (TCGA) and our ex vivo GBM tissues further showed that ARG1/ARG2 is variably expressed in patient specimens (Supplementary Figure S9) as well as in a mouse model of glioma (Supplementary Figure S10A and S10B, respectively). Interestingly, treated patients with higher expression of ARG2 tended to have shorter survival than those with lower ARG2 expression (Figure 6D, p=0.268). We observed a similar trend for reduced patient survival with higher median ARG2 expression, albeit it was not statistically significant (Supplementary Figure S11). ARG2 upregulation was also observed in two Gene Expression Omnibus (GEO) datasets (GSE2223 and GSE15824), but not in two others (GSE42656 and GSE31262), which is consistent with the heterogeneity of the disease (Figure 6E).

Our present study is limited by in vitro findings as there is yet no suitable mouse model for the study owing to the species-specific properties of HCMV and murine CMV, which bears little sequence homology with HCMV (Supplementary Figure S12) although several functional proteins homologs have been identified [21–23]. However, effort is ongoing and it is possible that suitable humanized mouse models to study HCMV pathogenesis will be available in the future as reviewed recently [24]. Nevertheless, our findings highlight the crucial role of ARG2 and its upregulation by HCMV in tumorigenesis and thus may identify an “Achilles heel” that could be exploited for therapeutic intervention.

## MATERIALS AND METHODS

### Viral stock and cells

The viral stock was a plaque-purified clinical isolate, VR 1814, from cervical secretions (a kind gift from Dr. Giuseppe Gerna, University of Pavia, Italy). The virus was propagated in human umbilical vein cells (HUVECs) [Clonetics, Lonza; or isolated from donors as described [25], titrated in MRC-5 fibroblasts (ATCC), aliquoted, and frozen at −80°C as described [6]. Alternatively, the virus was further passaged once in MRC-5 cells, which was subjected to a freeze-thaw cycle; the cellular materials were clarified and ultracentrifuged at 28,000 rpm for 1h at 4°C (Beckman, Optima L-90K Ultracentrifugation). The viral pellet was resuspended in 0.1 M sucrose-phosphate buffer to increase the viral titer as described [26]. The GBM cell lines U-373 MG (Uppsala), U-251 MG, and U-343 MGa (a kind gift from Dr. M. Nister, Karolinska Institutet, Stockholm, Sweden) were used for *in vitro* experiments. The growth medium for HUVECs or MRC-5 was as described before [6]. All GBM cell lines were grown in RPMI supplemented with 10% fetal bovine serum, 100 U/ml of penicillin, 100 μg/ml streptomycin, and 5 μg/ml plasmocin (InvivoGen). All cells were maintained in a humidified 5% CO_2_ incubator and verified as mycoplasma-free with the MycoAlert mycoplasma detection kit (Clonetics, Lonza).

### Plasmid and generation of stable cell line

The mammalian expression plasmid pcDNA 3.1(+)-ARG2 was generated by the GeneArt Plasmid Construction Service (Invitrogen). To generate stable cell lines, we adapted the method in Lonza's *Guideline for generation of stable cell lines*. In brief, we used Lipofectamine 2000 (Invitrogen) to transfect U-251 MG cells with ARG2 expression plasmid. Transfected cells harboring ARG2 plasmid were selected by limiting serial dilution in the presence of 1.5 mg/ml G418 (Geneticin, Invitrogen).

### Viral infection, RNA isolation, and TaqMan PCR

Approximately 3 × 10^5^ cells in 12-well plates were infected with HCMV at different MOI as described [6]. In brief, cells were lysed, total RNA was isolated, and cDNA was synthesized and analyzed by quantitative TaqMan PCR at 1, 3, and 5 days post-infection (dpi). TaqMan Fast Universal PCR Master Mix (Life Technologies) was used with the following primers/probes: ARG II (assay ID, Hs00982833_m1), MMP2 (assay ID, Hs01548727_m1), MMP9 (assay ID, Hs00234579_m1), human β2-microglobulin (B2M, assay ID, Hs00984230_m1), and 18S rRNA (cat. no. 4310893E) (Life Technologies). B2M or 18S rRNA was used for normalization [6]. TaqMan assays were performed as recommended by the manufacturer, using a 7900HT Fast Real-Time PCR system (Applied Biosystems) with a total cycle of 40 and final volume of 10 μl per reaction. The results were analyzed with SDS 2.4 software, and the 2^−ΔΔCt^ method was used to quantify relative expression.

### Short interfering RNA (siRNA) against ARG2, IE72, and IE86

The sequences of IE72 (IE233) and IE86 siRNA have been described [6] and were obtained from Invitrogen. siRNA against ARG2 was a Trilencer-27 siRNA (cat no. SR300277), siRNA negative control was a scrambled negative control duplex (cat. no. SR30004) (Origene Technologies). Transfection of siRNAs and infection protocols were essentially as described [6] and used the amount of Lipofectamine RNAiMAX reagent (Invitrogen) and siRNA recommended by the manufacturer (Invitrogen). In brief, cells at 80–90% confluency in 12-well plate were transfected with siRNA 24 h before infection with HCMV at an MOI of 5. The cells were harvested for quantitative TaqMan PCR analysis after 96 h of infection.

### Western blot analysis

The western blot assay was performed with 4–15% precast gels (Biorad). In brief, protein lysates were fractioned by SDS-PAGE, transferred onto PDVF membranes, blocked, and probed with monoclonal mouse anti-HCMV IE (Argene; 1:3000) and rabbit polyclonal anti-ARG2 (H-64, sc-20151, Santa Cruz Biotechnology; 1:500). After three washings in TBS containing 0.1% Tween buffer, the membranes were incubated with anti-mouse IgG (1:5000) or anti-rabbit IgG (1:5000) conjugated to horseradish peroxidase (both from Santa Cruz Biotechnology). Equal loading of proteins was verified with mouse monoclonal anti-β-actin (Thermo Scientific; 1:3000). Bound antibodies were detected with the ECL-Prime chemiluminescence kit (Amersham, GE Healthcare Life Sciences).

### Immunofluorescence (IF) and immunohistochemical (IHC) staining

IF on GBM cells and tissues as well as IHC on formalin-fixed paraffin-embedded (FFPE) sections were performed as described [6]. The FFPE sections were obtained from the Department of Pathology, Karolinska University Hospital. The primary antibodies were mouse anti-IE (Millipore, MAB810R; 1:500), mouse anti-CMV late antigens (Millipore, MAB8127, clone 1G5.2; 1:500), rabbit anti-ARG 2 (Santa Cruz Biotechnology, H-64, sc-20151; 1:500 for IF and 1:200 for IHC) and goat anti-alpha smooth muscle actin (α-SMA, Abcam, ab21027; used at 1:200 dilution). For dual IF, the secondary antibody was goat anti-mouse conjugated to Alexa Fluor 488 (Molecular Probes, Invitrogen, A11001; 1:500) or goat anti-rabbit conjugated to Alexa Fluor 594 (Molecular Probes, Invitrogen, A11012; 1:500); for triple IF, the secondary antibody was donkey anti-mouse conjugated to Alexa Fluor 488 (cat. no. A21202), donkey anti-rabbit conjugated to Alexa Fluor 594 (cat. no. 21207) and donkey anti-goat conjugated to Alexa Fluor 680 (cat. no. A21084) (All from Molecular probes and used at 1:500 dilution). For IHC, the secondary antibody was anti-mouse or anti-rabbit conjugated to HRP (Vector, ImmPRESS kit); immunoreactivity was revealed with DAB (Innovex Biosciences, NB314SBD) as the chromogen. All primary antibodies were incubated overnight at 4°C and added together for dual or triple IF staining where indicated. The immunoreactivity was revealed by adding secondary antibody conjugated to Alexa Fluor and analyzed with Zeiss confocal microscope LSM 700. As a control for IF, the primary antibody was replaced by TBS. For IHC, the frontal part of 10 normal aging brains was used as control to GBM tissue specimens.

The GBM tissue sections were from the Karolinska University Hospital (ethical number 2008/628-31/2), Sweden. The normal aging brains were from the Department of Pathology, University Malaya Medical Center, Malaysia (ethical number 896.7). The use of patient materials was approved by the ethics committees at both institutions, and the studies were conducted in accordance with the Declaration of Helsinki.

### MTT cell proliferation assay

Approximately 5 × 10^3^ cells were seeded onto 96-well plates and assayed for cellular metabolic activity at 1 or 3 days using MTT kit as recommended by the manufacturer (Roche). Absorbance was read at 570 nm with the reference wavelength at 670 nm.

### In vitro wound healing assay

The wound healing assay was adapted from Liang et al. [27]. In brief, approximately 3 × 10^5^ cells were seeded onto 12-well plates, grown to 95-100% confluency, scratched with 10–100-μl pipette tips, and rinsed twice with PBS and then replenished with growth medium or growth medium containing the arginase inhibitor N (omega)-hydroxy-nor-L-arginine (nor-NOHA) (a kind gift from Drs. Jiangning Yang and John Pernow) at a final concentration of 1 mM or 3 mM. At different time points after the scratch phase-contrast images were obtained with a 4× objective lens, and wound healing was assessed at five randomly selected points by measuring the width of the scratch, expressed as AU, using Adobe Photoshop CS5.

### Migration and invasion

Cell migration and invasion were assessed by using CytoSelect 24-well Cell Migration and Invasion Assay kit (cat no. CBA-100-C, Cell Biolabs), according to the manual. In brief, 1.5 × 10^5^ cells in 300 μl of RPMI were placed on an 8-μm pore size polycarbonate membrane insert (for migration) or on a polycarbonate membrane insert coated with a basement membrane layer (invasion assay) in 500 μl of 10% RPMI. After incubation for 24 h (migration) or 48 h (invasion assay), non-migratory cells in the upper chamber were carefully removed with cotton-tipped swabs, and the migratory cells were stained and read by a plate reader (Versamax tunable microplate reader, Molecular devices) at an optical density of 560 nm.

### Angiogenesis or tube-forming assay

The angiogenic capacity of cells was assessed with a tube forming assay as described [28], using reduced growth factor-Matrigel (BD Biosciences) in μ-Slides Angiogenesis (Ibidi). In brief, the optimal number of cells was seeded onto Matrigel-coated Ibidi slides and photographed at the indicated time. For treatment with nor-NOHA, U-251 MG-ARG2 was either mock-treated or treated with 1 mM or 3 mM nor-NOHA for 5 days before seeding onto Matrigel and photographed.

### ELISA

The activity of MMP2/MMP9 or Nitric Oxide Synthase (NOS) was measured using commercially available ELISA kits accordingly to the manufacturers' instruction. In brief, the Gelatinase (MMP-2/MMP-9) Activity Assay kit (cat. no. CBA003, Calbiochem) utilizes a triple-helical, collagen-like, fluorogenic substrate that is highly selective for MMP2 and MMP9 but not MMP1, MMP3, MMP13 and MMP14. The presence of MMP2 and/or MMP9 cleaves the fluorogenic substrate, resulting in an increased in fluorescence when excited at 320 nm and acquired at 405 nm. The supernatants were diluted with Activation Buffer in 1:3 and incubated at 37°C for 4h before reading with the WallacVictor^2^ 1420 Multilabel Counter (Perkin Elmer).

For the NOS activity, the indirect measurement of nitric oxide generated by NOS was measured at 540 nm (cat. no. K205-100, BioVision). The human VEGF was determined with Duoset ELISA accordingly to the manufacturer's protocol (cat. no. DY293B-05, R&D Systems).

### In silico analysis of TCGA and GEO datasets

Gene expression data in TCGA (https://tcga-data.nci.nih.gov/) for GBM are publicly available and firehose developed by the Broad Institute (http://gdac.broadinstitute.org) allows access to pre-processed mRNA expression data in which the median method was used for the standardization of the original GBM data (retrieved stddate 20141206). The data comprised 538 patient cases with median mRNA expression values which we further merged with clinical information, and then removed 13 patient cases because of unavailability of detailed annotations. The median of mRNA expression values for ARG2 of 525 patients was determined, following which we had to remove one patient where the survival information was not available. Survival was analyzed for treated (recurring or secondary GBM) (n=20) or untreated (primary *de novo* GBM) patients (n=498), with each group divided into patients with either greater than (>) or less than or equal to (≤) median ARG2 expression. Six patients denoted only as “GBM” in TCGA were excluded due to lack of information on primary/recurring status, thus could not be sorted into either of the above categories.

We also analyzed gene expression data for GBM available in GEO (Gene Expression Omnibus, http://www.ncbi.nlm.nih.gov/geo/) database. Data from four available patient GEO datasets, GSE2223 (normal=4, GBM=27), GSE15824 (normal=2, GBM=12), GSE31262 (normal=5, GBM=9) and GSE42656 (normal=4, GBM=5), were obtained for the ARG2 expression. Normal refers to normal brain tissue and GBM refers to brain tumor. The difference in expression levels of ARG2 in GBM compared to normal was shown using boxplot and, due to unequal sample sizes, the Welch's t-test was used to compute the statistical significance.

The R project for statistical computing (http://www.r-project.org/) was used for generating survival analysis plots.

### Statistical analysis

Data were analyzed by two-tailed *t* test, using Prism (version 5, GraphPad Inc.). One-way ANOVA was used to compare the effect of nor-NOHA treatment on gap closure and number of branches. P value <0.05 was considered significant. The R project for statistical computing (http://www.r-project.org/) was used for generating survival analysis plots.

## SUPPLEMENTARY FIGURES


