# Supplementary material for: Human cytomegalovirus may promote tumour progression by upregulating arginase-2
Source: Oncotarget. 2016 May 30;7(30):47221–31. doi: 10.18632/oncotarget.9722 (PMC5216936; doi:10.18632/oncotarget.9722)
Supplement: Supplementary file 1 [file oncotarget-07-47221-s001.pdf]

SUPPLEMENTARY FIGURES

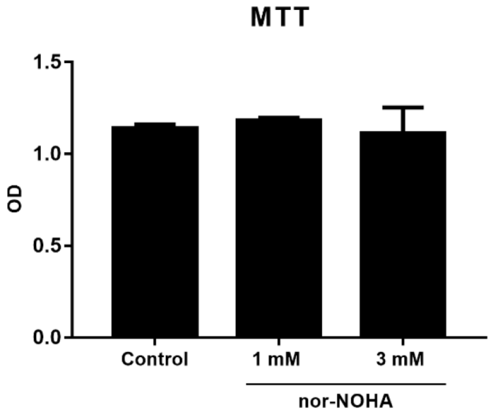

Supplementary Figure S1: Toxicity of different doses of ARG2 inhibitor, nor-NOHA, in U-251 MG cell line was evaluated using MTT assay. OD, optical density. Bars represent mean±SD (n=10).

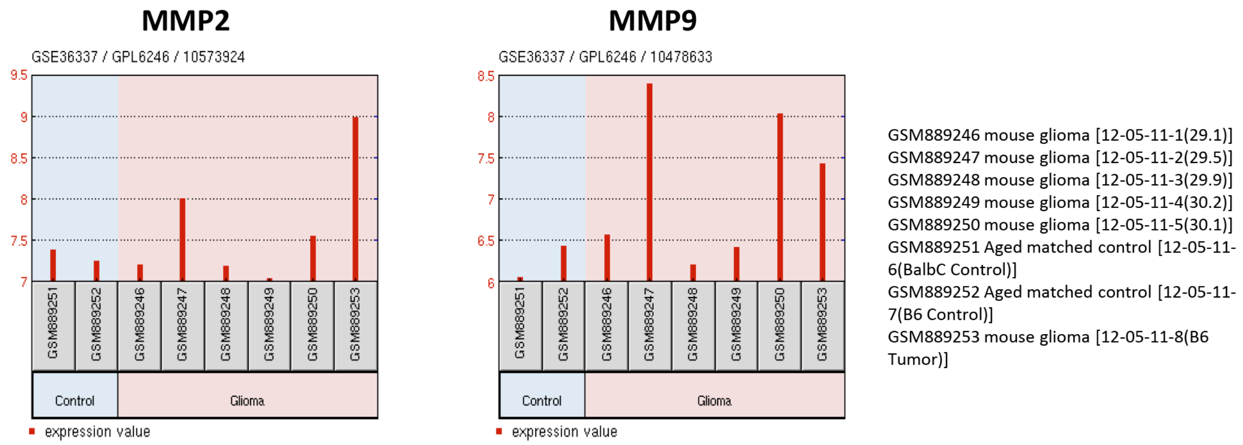

Supplementary Figure S2: Expression of MMP2 and MMP9 in the mouse model of glioma.

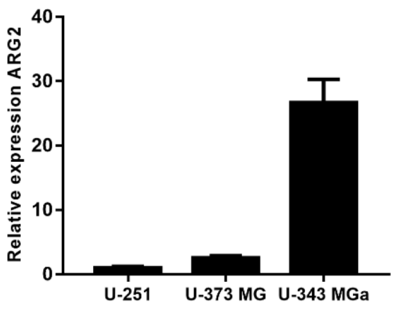

Supplementary Figure S3: Relative expression of ARG2 in different GBM cell lines was determined by qPCR and normalized to U-251 MG. Beta 2-microglobulin was used as endogenous control. Bars represent mean±SD (n=3).

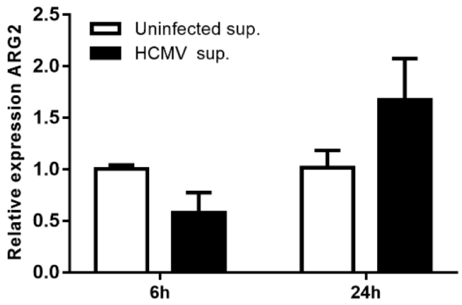

Supplementary Figure S4: Relative expression of ARG2 in U-251 MG cells cultured with supernatants from uninfected or HCMV infected cells for 6h or 24h. Bars represent mean±SD (*n*=3).

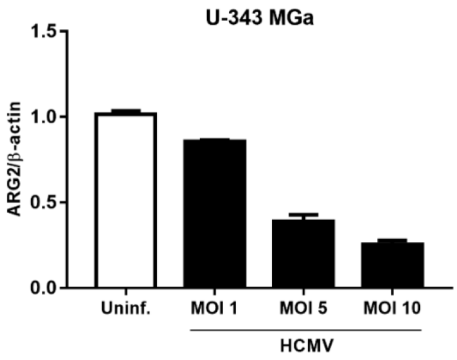

Supplementary Figure S5: Quantification of ARG2 protein levels in U-343 MGa cells infected with increasing MOI of HCMV determined by western blot shown in Figure 4. Bars represent mean±SD (*n*=2).

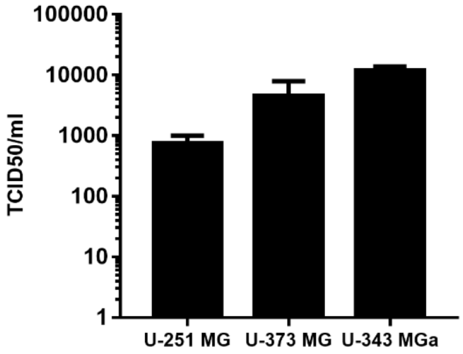

Supplementary Figure S6: Titration of HCMV virions produced in different GBM cell lines infected with HCMV using TCID50 method. Bars represent mean±SD (*n*=3).

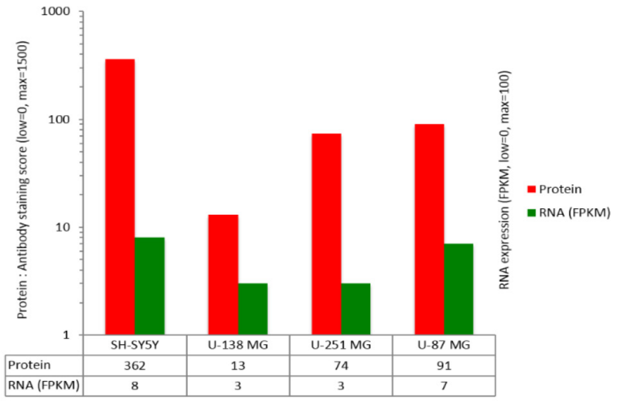

Supplementary Figure S7: Relative expression of ARG2 in different GBM cell lines from the Protein Atlas.

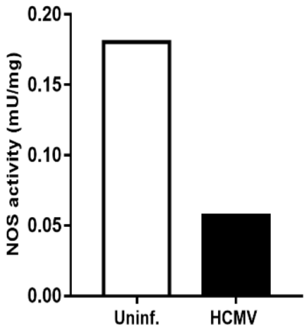

Supplementary Figure S8: NOS activity was detected in U-251 MG uninfected or infected with HCMV at 5dpi. Data represents mean (n=1).

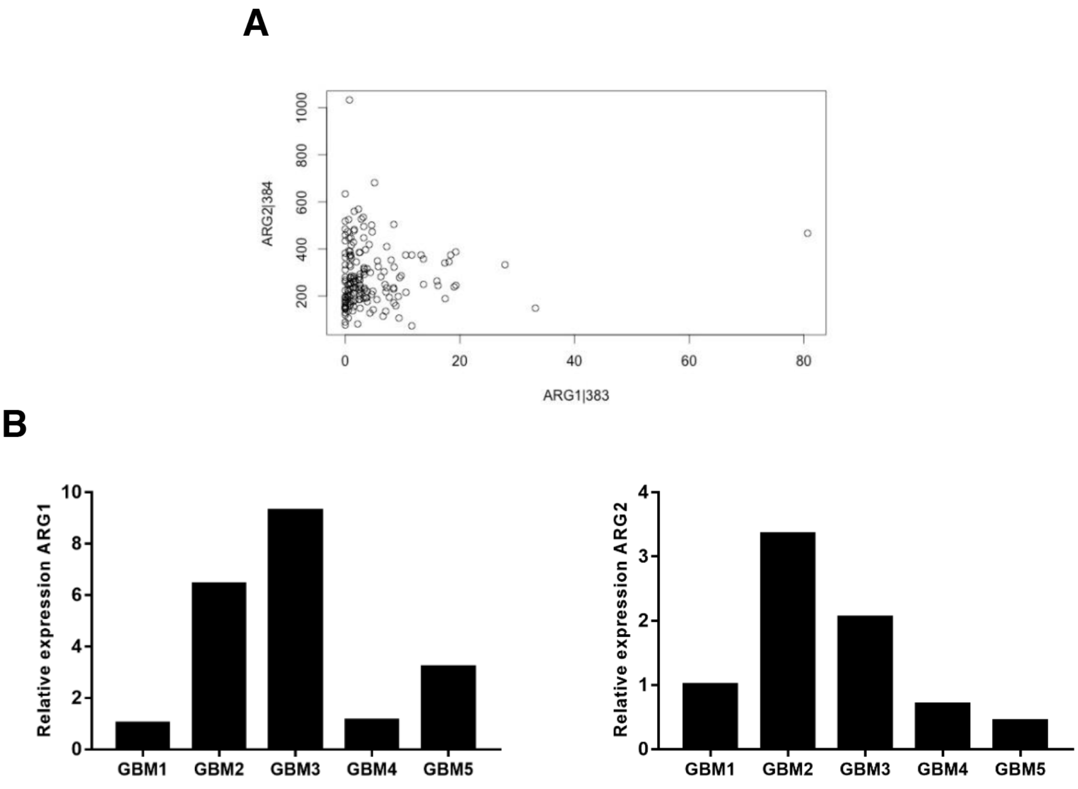

Supplementary Figure S9: A. ARG1 vs ARG2 RNA expression in 166 GBM patients from TCGA database. The Entrez gene ID are 383 and 384. Axis x and y represent RSEM (RNA-seq by Expectation-Maximization) **B.** Expression levels of ARG1 (left) and ARG2 (right) in different GBM tissues was determined by qPCR and normalized to GBM1 tissue.

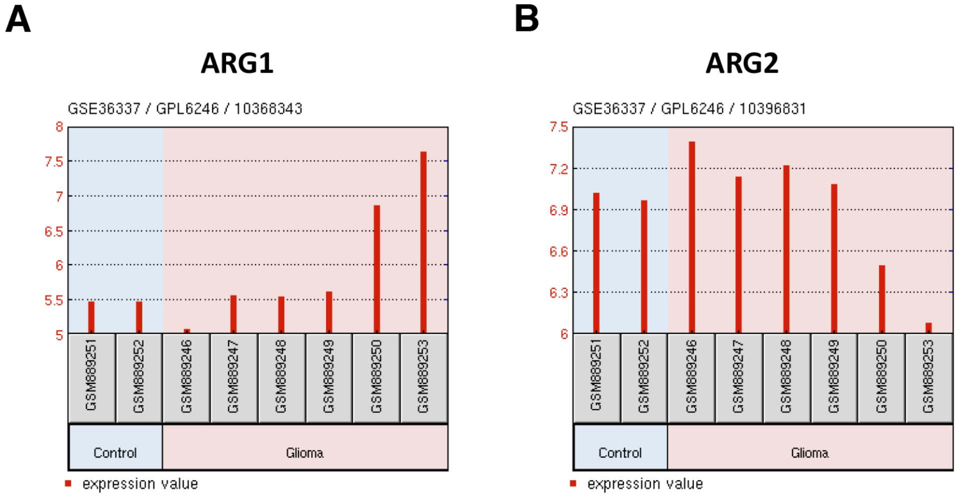

Supplementary Figure S10: Expression of ARG1. A. and ARG2 **B.** in the mouse model of glioma.

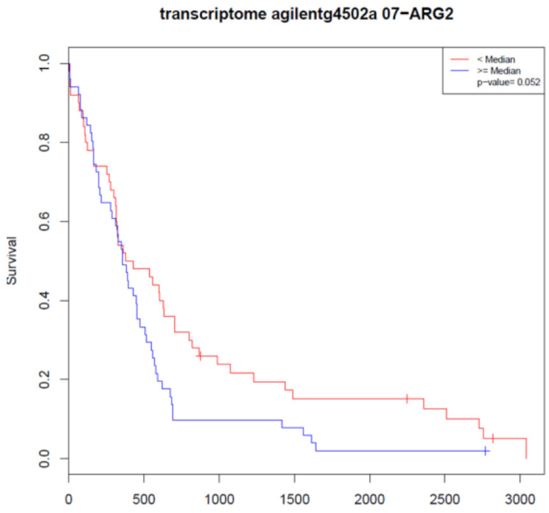

Supplementary Figure S11: Correlation between ARG2 expression and survival at transcriptome analysis in GBM patients using data from TCGA database.

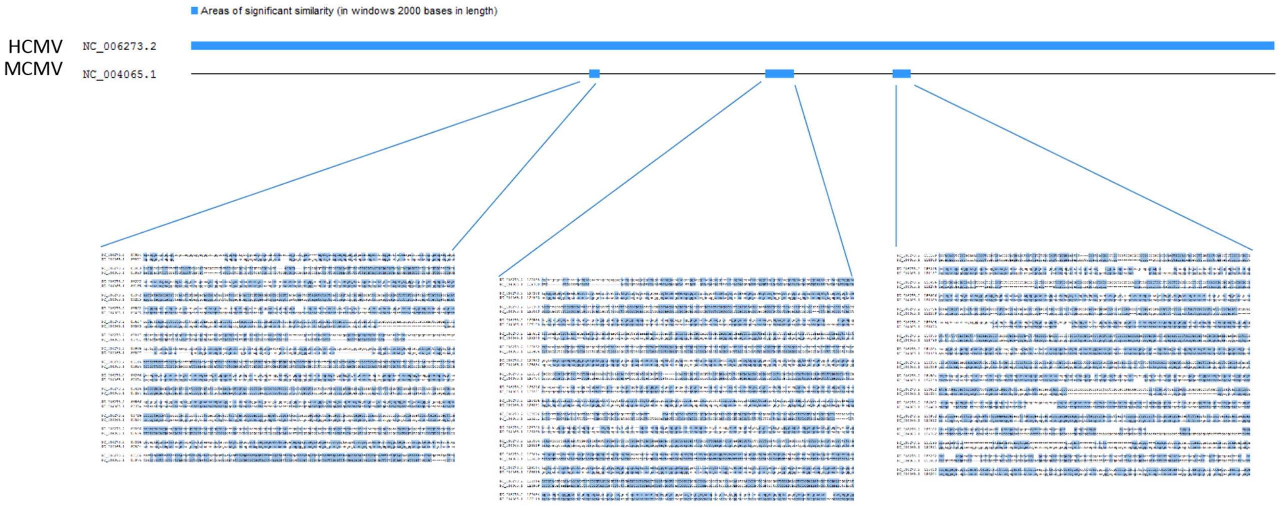

Supplementary Figure S12: Whole genome alignment of both reference sequences of murine and human cytomegalovirus (MCMV and HCMV, respectively). Blue box represents areas of significant similarity.
